# Genome-Wide Identification of *CaPLATZ* Family Members in Pepper and Their Expression Profiles in Response to Drought Stress

**DOI:** 10.3390/genes16060632

**Published:** 2025-05-24

**Authors:** Xingliang Wang, Yue Huang, Na Yang, Xue Wang, Yuanqian Wang, Wenyao Ma, Hui Zhang

**Affiliations:** 1 Horticultural Branch of Heilongjiang Academy of Agricultural Sciences, Harbin 150069, China; yyfylajiao@163.com (X.W.); 18246888551@163.com (Y.H.); wangxue2025a@163.com (X.W.); wyq046535@163.com (Y.W.); linzhiiii@163.com (W.M.); 2 Harbin Academy of Agricultural Sciences, Harbin 150029, China; 13789700617@163.com

**Keywords:** pepper, bioinformatics, *PLATZ* family, drought stress

## Abstract

**Background:** The plant AT-rich sequence and zinc binding (PLATZ) transcription factors constitute a zinc-dependent protein family implicated in various developmental processes and responses to abiotic stress. Nevertheless, comprehensive investigations on *PLATZ* gene functions in pepper (*Capsicum annuum*) have not been extensively performed. **Methods:** In the present study, bioinformatics methods coupled with quantitative real-time PCR (qRT-PCR) were employed to characterize the phylogenetic relationships, chromosome distribution, structural composition, cis-regulatory elements, evolutionary dynamics, and expression responses of *CaPLATZ* genes under drought stress conditions. **Results:** Phylogenetic analyses categorized the *CaPLATZ* genes into four distinct subgroups, each exhibiting similar gene structures and conserved motif patterns within its subgroup. A total of 11 *CaPLATZ* genes were nonuniformly located across eight pepper chromosomes, and synteny analyses identified a duplication event involving a single gene pair. The assessment of cis-acting regulatory elements indicated potential involvement of *CaPLATZ* genes in responses to abiotic stresses and various phytohormones. Furthermore, qRT-PCR results revealed differential expression of most *CaPLATZ* genes under drought-induced stress. **Conclusions:** Collectively, these findings support the functional roles of *CaPLATZ* transcription factors in mediating developmental processes and enhancing drought tolerance in pepper.

## 1. Introduction

Regulation of gene expression by transcription factors is a fundamental mechanism by which plant development programs are modulated and plant responses to environmental stressors are achieved adaptively. Transcription factors act by binding specifically to given cis-acting elements upstream of target gene promoters to either activate or repress transcription [1]. Among plant transcription factor families, zinc finger proteins form a notably large group implicated in several biological activities such as morphogenesis and transcriptional regulation, as well as in stress responses, as seen in *WRKY* and *ZAT* proteins [2]. The plant-specific *PLATZ* subfamily, originally identified in pea (*Pisum sativum*) as *PLATZ1*, contains zinc-binding proteins, which are AT-rich sequences nonspecifically interacting and acting as transcriptional repressors in a primary capacity [3]. Computational structure analyses have identified two strongly conserved zinc finger domains in *PLATZ* proteins: an N-terminal motif (C-x^2^-H-x^10^-C-x^2^-C-x^4–5^-C-x^2^-C-x^3–7^-H-x^2^-H) and a central region motif (C-x^2^-C-x^10–11^-C-x^3^-C). Both motifs require zinc ions for performing their activities in binding to DNA [4].

Research on the *PLATZ* gene family has increased significantly in recent years, where a number of members of the *PLATZ* gene family have been identified in various plant species, such as *Solanum lycopersicum* [5], *Triticum aestivum* [6], *Malus* [7], *Ginkgo biloba* [8], and *Glycine max* [9]. A number of studies have addressed functions of *PLATZ* transcription factors in regulating plant growth and development. In *Arabidopsis thaliana*, *AtPLATZ3* has a function of controlling leaf growth and senescence-like responses [10]. Furthermore, *AtPLATZ7* plays a key role in regulating the development of the *Arabidopsis* RAM by mediating root meristem growth factor 1 (*RGF1*) signaling [11]. *VviPLATZ1* plays a critical role in controlling female flower morphology in grapes, with loss-of-function mutants exhibiting reflex stamens [12]. In rice, the *PLATZ* transcription factor *GL6* promotes increased grain length through facilitating cell proliferation within the developing caryopses and panicles [13]. The soybean *GmPLATZ* protein presumably functions by directly interacting with cyclin and *GmGA20OX* gene promoters, thereby stimulating gene expression and subsequent cell proliferation [14].

Drought is a major abiotic stress factor that severely impacts global agricultural production, driving significant research efforts to develop drought-tolerant crops. Emerging evidence highlights the critical roles of *PLATZ* transcription factors in regulating plant responses to drought stress conditions. For example, in *A. thaliana*, *AtPLATZ1* increases dehydration tolerance in vegetative tissues [15]. *AtPLATZ4* targets the promoter of the plasma membrane aquaporin *PIP2;8*, inhibiting its expression and enhancing drought resistance [16]. Overexpression of *PhePLATZ1* enhances drought stress resistance in transgenic plants by modulating osmotic balance, improving water retention, and reducing membrane and oxidative damage [17]. Ectopic expression of cotton-derived *GhPLATZ1* in transgenic *Arabidopsis* plants confers enhanced tolerance to osmotic stress induced by salt and mannitol treatments, thereby accelerating seed germination and seedling establishment [18].

Pepper (*C. annuum* L.) is a significant vegetable crop, valued for its nutritional and economic importance. Pepper cultivation in China accounts for about 8–10% of the total vegetable planting area, contributing an output value of approximately 250 billion yuan. This makes pepper the leading vegetable in terms of both planting area and economic value. Throughout its growth period, pepper is often exposed to a variety of abiotic and biotic stresses, including drought, cold temperatures, insufficient illumination, pests, and pathogens [19]. Members of the *PLATZ* gene family play critical roles in orchestrating plant developmental pathways and adaptive responses to environmental stresses. However, research on PLATZ transcription factors has primarily concentrated on model plant systems, and comprehensive studies in pepper have not yet been reported. This study systematically identified and characterized the pepper *PLATZ* family, including analyses of their evolutionary history, chromosomal distribution patterns, gene structures, cis-regulatory elements, phylogenetic relationships, and drought-responsive expression profiles. The results provide valuable theoretical insights into *CaPLATZ* gene functions and present a molecular basis for breeding drought-tolerant pepper cultivars.

## 2. Materials and Methods

### 2.1. Genomic Identification of CaPLATZ Genes in Pepper

The complete genomic sequence of pepper cultivar Zunla-1 (*C. annuum* L.) was selected as a reference, with genomic data sourced from the Sol Genomics Network database [20]. Initially, *Arabidopsis PLATZ* proteins served as queries to conduct a homology search against the pepper protein dataset using the BlastP (NCBI-BLAST v2.10.1+) algorithm. Subsequently, the Hidden Markov Model (HMM) profile of *PLATZ* domains (PF04640.17) was retrieved from the Pfam database (https://pfam.xfam.org/) and utilized in hmmsearch analyses (HMMER v3.0), applying an E-value cutoff below 1 × 10^−5^. Candidate *CaPLATZ* proteins were ultimately identified through combined domain analyses using SMART (http://smart.embl.de/) and the Conserved Domain Database (CDD, https://www.ncbi.nlm.nih.gov/cdd/?term=, accessed on 22 May 2025), retaining only sequences harboring confirmed *PLATZ* structural domains. Eleven *CaPLATZ* genes were identified in pepper. These genes were systematically renamed as *CaPLATZ1*–*CaPLATZ11* according to their chromosomal location. Online tools were employed to predict the physicochemical properties of the *CaPLATZ* proteins. Subcellular localization predictions for these proteins were performed using the WoLF PSORT server (https://wolfpsort.hgc.jp/).

### 2.2. Phylogenic Analysis

Based on the work of Kumar et al. [21], the ClustalW (ClustalW v2.0.11) method within the MEGA-X ((MEGA v10.2)) software was used to run multiple sequence alignments of pepper and *Arabidopsis CaPLATZ* amino acid (AA) sequences. After that, we used the maximum likelihood (ML) technique to build a phylogenetic tree, and we tested the reliability of each node using bootstrap analysis with 1000 replications. All the sequences used in the phylogenetic analysis are provided in Table S1.

### 2.3. Analysis of Synteny and Homologous Gene Pairs

Syntenic comparisons among the *A. thaliana*, *S. lycopersicum*, and *Oryza sativa* genomes were carried out using TBtools software (TBtools v2.225) [22]. Genomic sequences for these comparative species were obtained from the Ensembl Plants database (https://plants.ensembl.org/).

### 2.4. Analysis of CaPLATZ Gene Structures and Phylogenetics

By leaving all other parameters at their default values, MEME Suite was able to identify conserved motifs in the pepper *PLATZ* gene family, with a target of 10 unique motifs. Genome annotation files in the GFF3 format were used to extract the exon–intron architecture of *CaPLATZ* genes. With 1000 bootstrap repeats for support, phylogenetic reconstruction was carried out using the ML technique in MEGA-X, and ClustalW was used to build multiple sequence alignments.

### 2.5. Identification of Cis-Regulatory Elements in CaPLATZ Genes

We used the PlantCARE database (http://bioinformatics.psb.ugent.be/webtools/plantcare/html/) to systematically identify cis-acting regulatory elements (CAREs), and we recovered promoter sequences (2000 bp upstream) for all eleven *CaPLATZ* genes from the genomic dataset [23]. In order to facilitate complete biological data analysis, the discovered CAREs were then visually represented using TBtools software [22].

### 2.6. Plant Materials and Stress Treatment Protocols

A growing substrate that consisted of vermiculite, peat, and perlite in a proportion of 1:3:1 (*v*/*v*/*v*) was used to nurture pepper seedlings in plastic containers with dimensions of 10 cm × 10 cm × 10 cm, with each container housing one seedling. The seedlings were cultivated in a glasshouse under carefully regulated conditions, with temperatures maintained at 28 °C during the day and 22 °C during the night, along with a relative humidity of 75%. To conduct osmotic stress treatments, we exposed seedlings to a 400 mM D-mannitol solution in a liquid medium when they were uniformly four leaves old. At 0, 6, 12, and 24 h after treatment, data were obtained from both the control and treated groups. The freshly picked leaves were promptly placed in liquid nitrogen for flash freezing and kept at a temperature of −80 °C. For each condition in the experiment, six seedlings were used.

### 2.7. Expression Analysis for CaPLATZ Genes

The transcriptional patterns of *PLATZ* family members were examined utilizing public transcriptomic datasets GSE45037 (https://www.ncbi.nlm.nih.gov/) and PepperHub (http://lifenglab.hzau.edu.cn/) [20,24]. Visualization of gene expression differences was accomplished by generating heatmaps with TBtools software. To validate gene expression under drought stress conditions, total RNA was extracted from pepper tissues exposed to drought stress using a RNAprep Pure Plant Kit (TianGen, Beijing, China). Reverse transcription into complementary DNA was subsequently performed with a KOD One™ PCR Master Mix reverse transcription kit (TOYOBO, Shanghai, China). Quantitative real-time PCR (qRT-PCR) was conducted using an ABI QuantStudio 3 system (Applied Biosystems, Waltham, MA, USA) and 96-well reaction plates under the following parameters: initial denaturation at 95 °C for 3 min, 40 cycles of denaturation at 95 °C for 10 s, and annealing/extension at 68 °C for 15 s. Relative expression of target genes was calculated according to the 2^−ΔΔCT^ method, and primer details are listed in Supplementary Table S2.

## 3. Results

### 3.1. Identification of CaPLATZ Members in Pepper

**Figure 1:**
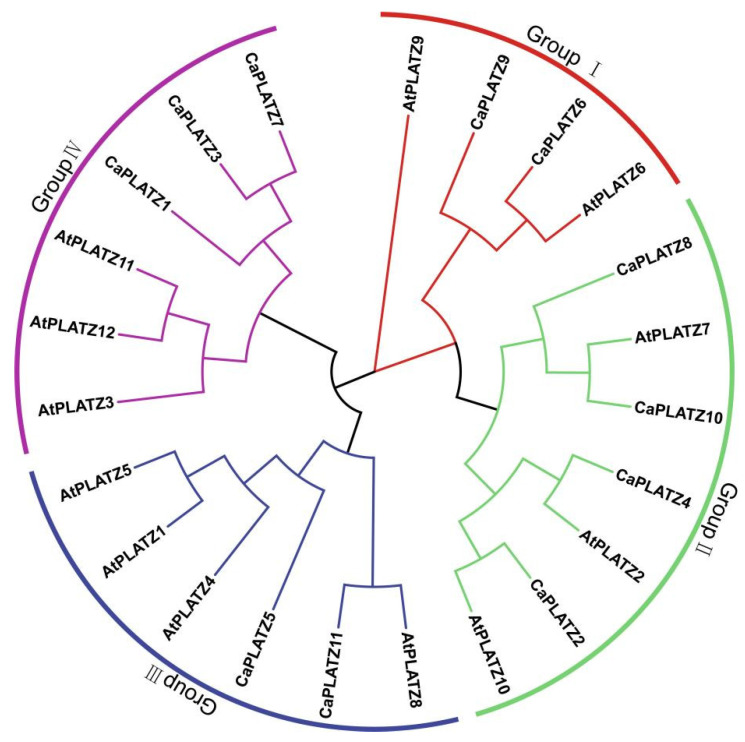
Phylogenetic trees depicting *PLATZ* family members in *A. thaliana* and pepper. The *PLATZ* proteins were classified into 4 groups distinguished by different colors.

Through iterative homology searches using the *PLATZ* domain (Pfam: PF04640) as a query combined with BlastP analysis, eleven *CaPLATZ* genes were identified in the pepper genome. Subsequent analyses characterized these *CaPLATZ* proteins based on chromosomal distribution, AA length, molecular weight (MW), and theoretical isoelectric points (pI) (Table 1). Uneven distribution of the *CaPLATZ* genes was observed across chromosomes 1, 2, 4, 5, 6, 7, 9, and 10. The length of *CaPLATZ* proteins ranged from 174 AAs (*CaPLATZ6*) to 247 AAs (*CaPLATZ10*). Additionally, the MWs ranged between 20,057.15 Da (*CaPLATZ6*) and 28,113.13 Da (*CaPLATZ10*), and their theoretical pI varied from 6.16 (*CaPLATZ4*) to 9.73 (*CaPLATZ3*). A phylogenetic tree constructed from *CaPLATZ* and *AtPLATZ* sequences classified these proteins into four distinct groups (Figure 1).

### 3.2. Duplication Modes and Collinearity Analysis of the CaPLATZ Gene Family in Pepper

**Figure 2:**
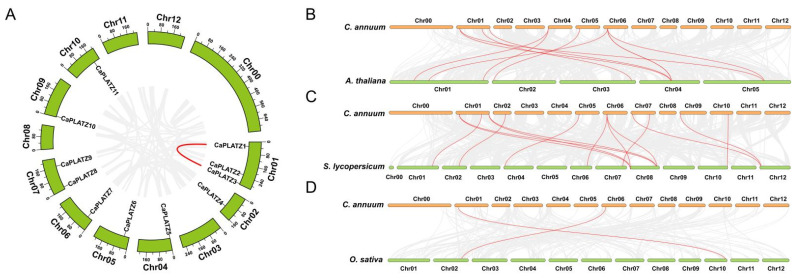
Chromosome-level analyses of the *CaPLATZ* gene family in the pepper genome assembly. (**A**) Chromosomal positions and synteny relationships among *CaPLATZ* genes. Red lines indicate syntenic gene pairs. (**B**–**D**) Synteny analyses comparing pepper *PLATZ* genes with those of *A. thaliana*, *S. lycopersicum*, and *O. sativa*, respectively.

Gene families typically arise via tandem duplications or large-scale segmental duplications during evolution. Segmental duplication analysis showed significant similarity between *CaPLATZ1* and *CaPLATZ3*, suggesting intra-chromosomal or segmental duplication events (Figure 2A). To further explore the evolutionary dynamics of *PLATZ* genes, synteny analyses among pepper, *A. thaliana*, *S. lycopersicum*, and *O. sativa* were conducted. The analysis identified eight pairs of homologous *PLATZ* genes between pepper and *A. thaliana*, nine pairs between pepper and *S. lycopersicum*, and only two pairs between pepper and *O. sativa* (Figure 2B–D).

### 3.3. Gene Structure Analysis of the CaPLATZ Genes in Pepper

**Figure 3:**
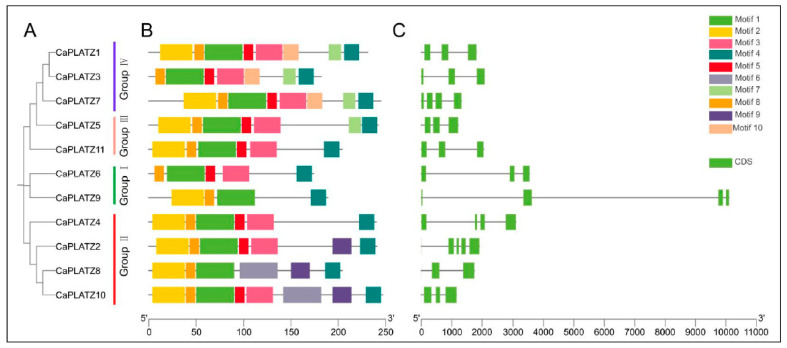
Conserved motifs and gene structures of the *CaPLATZ* gene family in pepper. (**A**) Phylogenetic relationships among the *CaPLATZ* proteins. (**B**) Motif composition patterns in the *CaPLATZ* proteins. (**C**) Exon–intron structural variations of the *CaPLATZ* genes.

Based on phylogenetic relationships, the 11 *PLATZ* proteins were categorized into four distinct groups, aligning with the earlier classification results (Figure 3A). The MEME suite analysis identified ten distinct conserved motifs (motifs 1–10) within the pepper *CaPLATZ* protein family (Table 2). These motifs, comprising 11–41 AAs, showed varying degrees of conservation among the groups. Specifically, motifs 1, 4, and 8 were conserved across all the *CaPLATZ* proteins. Moreover, the motif distribution was highly similar among the *CaPLATZ* proteins belonging to the same phylogenetic group, reflecting group-specific motif conservation (Figure 3B). Exon–intron structure analysis revealed substantial variation among the *CaPLATZ* genes; notably, *CaPLATZ8* contained only two exons, while the majority included three or four exons (Figure 3C). Such structural diversity likely contributes to the functional specialization of members within the *PLATZ* gene family.

### 3.4. Analysis of Cis-Acting Elements in CaPLATZ Promoter Regions in Pepper

**Figure 4:**
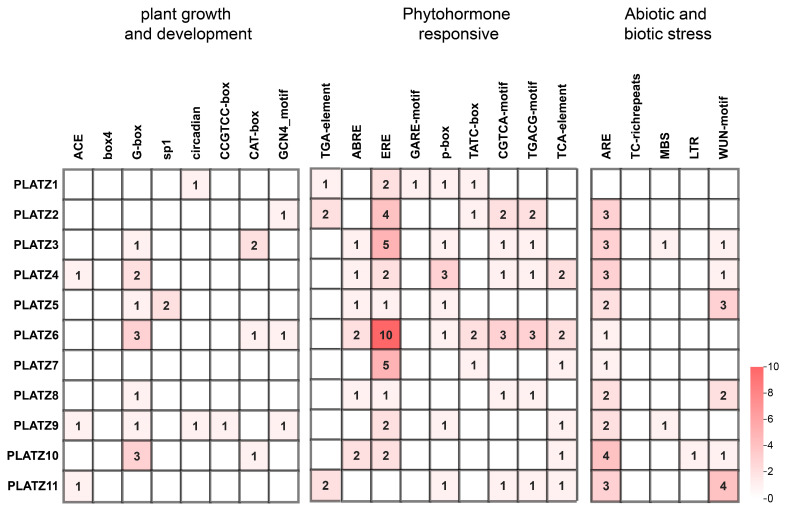
Distribution of CAREs in *CaPLATZ* gene promoters in pepper.

To investigate potential regulatory roles of *CaPLATZ* genes, a total of 154 CAREs were detected within the 2000 bp promoter sequences upstream of the identified genes. These CAREs were grouped into three functional classes associated with plant growth/development, hormonal signaling pathways, and abiotic stress responsiveness. Of particular significance was the abundance of elements responsive to stress stimuli and hormonal regulation. Notably, ethylene-responsive elements (EREs) were particularly enriched in *CaPLATZ* gene promoters, indicating their possible involvement in ethylene-mediated signaling cascades (Figure 4). These observations suggest complex regulatory networks involving multiple cis-elements that govern *CaPLATZ* expression in developmental processes and adaptive stress responses.

### 3.5. Expression Analysis of the CaPLATZ Genes in Pepper

**Figure 5:**
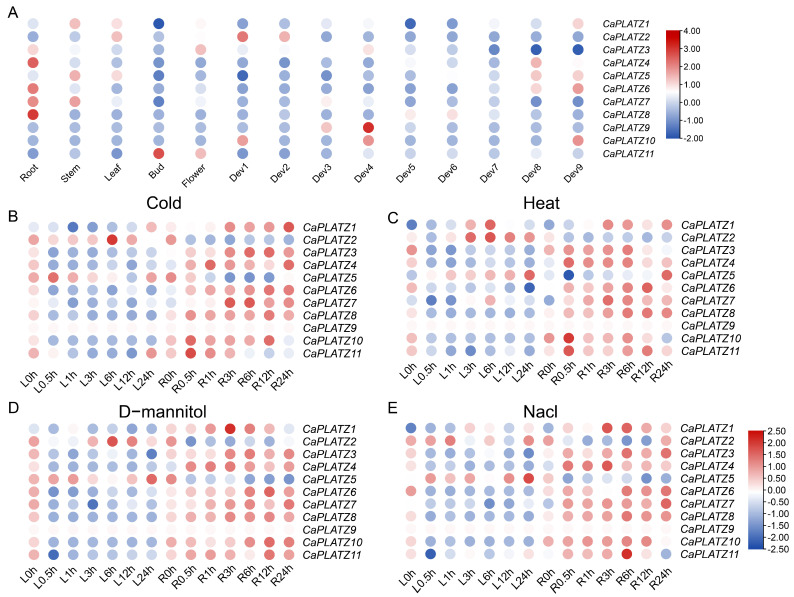
Expression patterns of the *CaPLATZ* gene family across different tissues and abiotic stresses. (**A**) Heatmap illustrating *CaPLATZ* expression in pepper tissues, including root, stem, leaf, bud, flower, and fruit developmental stages. The 1–9 stages of fruit development are presented in the five early stages of color breaking (0–1, 1–3, 3–4, and 4–5 cm long fruits and mature-green fruits), the color breaking stage (fruit beginning to turn red), and three late stages of color breaking (3, 5, and 7 days after color breaking), respectively. (**B**–**E**) Expression profiles under abiotic stresses: (**B**) cold stress, (**C**) heat stress, (**D**) drought stress, and (**E**) salt stress. Abbreviations: R, roots; L, leaves; Dev., developmental stages.

To elucidate the comprehensive expression patterns of the *CaPLATZ* genes across different tissues and under various environmental stresses, RNA-seq datasets from public resources and the Pepper Hub were examined, and the resulting profiles were visualized using heatmap analysis (Figure 5). Most *CaPLATZ* genes were expressed widely but displayed considerable variation across different tissues and developmental stages. Specifically, *CaPLATZ8* exhibited root-specific expression, whereas *CaPLATZ4*, *CaPLATZ9*, and *CaPLATZ10* showed extremely low or undetectable expression in all the tested tissues. Further analysis was conducted to investigate the response of the *CaPLATZ* genes to various abiotic stresses. Under different stress treatments in root tissues, significant upregulation of *CaPLATZ1*, *CaPLATZ3*, and *CaPLATZ7* was observed, with *CaPLATZ1* notably responding to D-mannitol and NaCl stresses. Additionally, *CaPLATZ5* exhibited marked induction in both leaves and roots under high-temperature conditions, suggesting involvement in abiotic stress responses.

### 3.6. Expression Patterns of CaPLATZ Genes Under Drought Stress

**Figure 6:**
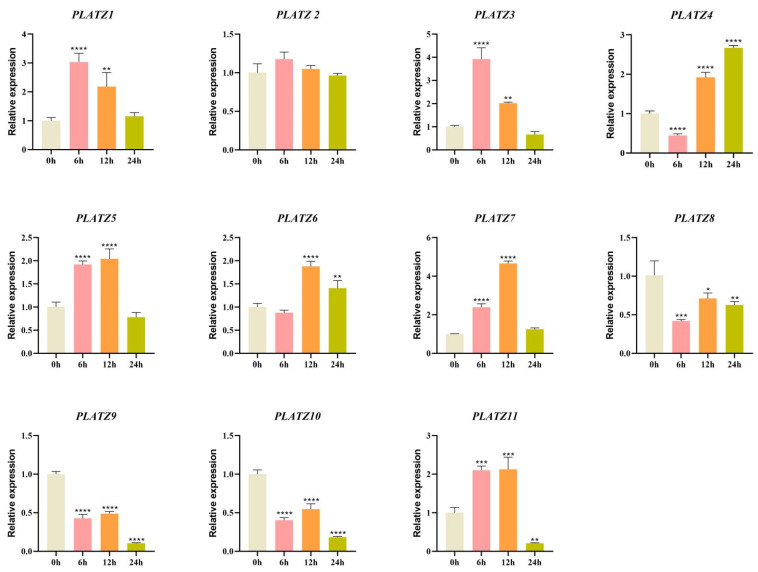
Validation of *CaPLATZ* gene expression under drought stress conditions using qPCR. Statistical significance levels: * *p* ≤ 0.05; ** *p* ≤ 0.01; *** *p* ≤ 0.001; **** *p* ≤ 0.0001 (*t*-test).

We performed qRT-PCR analyses to confirm RNA-seq-derived expression patterns of *CaPLATZ* genes during drought stress. The transcription levels of *CaPLATZ1*, *CaPLATZ3*, and *CaPLATZ7* significantly increased following drought treatment. The rapid induction of *CaPLATZ1* and *CaPLATZ3* (peaking at 6 h) likely reflects their role in early drought signaling. Their subsequent decline at 12 h may indicate feedback inhibition or prioritization of other stress-adaptive mechanisms. In contrast, the sustained upregulation of *CaPLATZ7* suggests its involvement in long-term drought adaptation. Moreover, *CaPLATZ4* and *CaPLATZ6* showed decreased expression at 3 h, which subsequently increased after 6 h of treatment (Figure 6). These findings indicate that different *CaPLATZ* members may participate in diverse regulatory pathways responding to drought stress.

## 4. Discussion

*PLATZ* family genes are zinc-dependent DNA binding proteins that have been proven to be involved in orchestrating plant developmental pathways and adaptive responses to environmental stresses [3]. Research on the *PLATZ* gene family has increased significantly in recent years, and a number of members of the *PLATZ* gene family have been identified in various plant species. In this study, we discovered 11 *CaPLATZ* genes based on the pepper genome sequence. The number of *PLATZ* gene family members in pepper was comparable to that in *Citrullus lanatus* (12) and *Hordeum vulgare* (11), but lower than in *Medicago sativa* (55) and *Linum usitatissimum* (28) [25,26,27,28]. Notably, the pepper genome (3.48 Gb) is 3.7 times larger than that of *M. sativa* (810 Mb) [29] and 10.04 times the size of that of *L. usitatissimum* (302 Mb) [30], suggesting no direct correlation between genome size and the number of *PLATZ* gene family members. Phylogenetic analysis delineated four distinct groups of *CaPLATZ*, aligning with findings in soybean [9]. Inter-chromosomal segment duplication and tandem duplication represent significant driving forces in the evolution of plant genomes and genetic systems [31]. Through chromosomal mapping and synteny analysis, a pair of segmentally duplicated genes was identified, indicating that segmental duplication mechanisms were primarily responsible for the expansion of the *PLATZ* gene family. Furthermore, the collinearity analysis revealed intimate evolutionary relationships among the PLATZ family genes in tomato and pepper, aligning with findings in *S. lycopersicum* [32], thus indicating similar functions of these gene families within the Solanaceae family. Analyzing gene structures and conserved motifs is essential in studying gene family evolution [33]. The analysis results revealed that the gene structures and motif distribution were notably consistent among *CaPLATZ* proteins within the same phylogenetic group, while variations were observed among different groups. These results indicated that structural and motif differences in the *CaPLATZ* gene family may confer functional diversity in the regulation of plant growth.

CAREs are important transcriptional regulators that adjust a wide range of biological phenomena, including developmental regulation, as well as responses to environmental stressors [34]. From the analysis of promoter sequences, several *CaPLATZ* genes were identified as having a number of hormone-signaling, as well as stress response, elements in this analysis. Ten *CaPLATZ* genes contained the element ERE, suggestive of a potential role in modulating mechanisms of adaptation to ethylene-mediated stress mechanisms [35]. Antioxidant-responsive elements (AREs) were also identified in ten *CaPLATZ* genes, strongly correlating with plant stress responses [36]. The existence of such regulatory elements as ERE constitutes structural evidence of *CaPLATZ* gene involvement in stress-related signaling mechanisms, providing insight into potential mechanisms of stress tolerance in pepper.

In this study, the majority of *CaPLATZ* genes exhibited significant expression in pepper roots, suggesting their potential involvement in root growth and development. Similarly, tomato *SlPLATZ17* showed elevated expression levels in root and floral tissues [5]. Among the *Malus MdPLATZ* genes (*MdPLATZ1*, *MdPLATZ2*, *MdPLATZ4*, *MdPLATZ6*, *MdPLATZ7*, *MdPLATZ11*, and *MdPLATZ19*), higher expression levels were observed in roots, consistent with tissue-specific expression analyses performed here [7]. These findings collectively indicate that *PLATZ* genes are highly expressed in roots across diverse plant species, implying a conserved functional role in root development.

Drought stress is a major factor affecting plant growth and development, which limits agricultural production [37]. To investigate the functional role of *CaPLATZ* genes in pepper’s response to drought stress, we analyzed their expression patterns under drought conditions. The transcription levels of *CaPLATZ1*, *CaPLATZ3*, and *CaPLATZ7* increased significantly following drought treatment. Specifically, *CaPLATZ1* and *CaPLATZ3* expression peaked at 6 h post-treatment but declined sharply by 12 h. In contrast, *CaPLATZ7* expression exhibited a sustained increase under prolonged drought stress. For example, overexpression of *AtPLATZ1* enhanced drought tolerance in transgenic *Arabidopsis* [18]. In tomato, the expression of *SlPLATZ17* was significantly upregulated during the initial phase of three independent stress treatments, reaching a peak at 1.5 h post-induction, followed by a gradual decline, though remaining consistently higher than pretreatment levels throughout the experimental period. Furthermore, *SlPLATZ17* silencing markedly reduced plant resistance to both salinity and drought stresses. Notably, this expression pattern aligns with those observed for *CaPLATZ1* and *CaPLATZ3* in related species, strongly suggesting conserved functional roles in abiotic stress adaptation among these PLATZ family members [5]. Similarly, overexpression of *GmPLATZ17* in soybean increased drought sensitivity and suppressed stress-related gene transcription, whereas silencing *GmPLATZ17* improved drought tolerance in transgenic soybean hairy roots [9]. Collectively, these findings suggest that *CaPLATZ* genes may contribute to pepper’s drought stress response, meriting further investigation.

## 5. Conclusions

In this study, 11 *CaPLATZ* genes were non-uniformly located across eight pepper chromosomes, and synteny analyses identified a duplication event involving a single gene pair. Phylogenetic analyses categorized the *CaPLATZ* genes into four distinct subgroups, each exhibiting similar gene structures and conserved motif patterns within its subgroup. The assessment of cis-acting regulatory elements indicated potential involvement of *CaPLATZ* genes in responses to abiotic stresses and various phytohormones. The qRT-PCR results showed that the transcription levels of *CaPLATZ1*, *CaPLATZ3*, and *CaPLATZ7* significantly increased following drought treatment. These results indicated that these genes are particularly critical in drought response. Overall, these findings provide insight into *CaPLATZ* gene evolution and form a worthwhile foundation for future breeding programs focusing on increased stress resistance in pepper.

## Figures and Tables

**Table 1:** Description of pepper *PLATZ* genes.

Number	Gene ID	Gene Name	Chr	Start	Stop	Strand	Size (aa)	MWs (Da)	pI	Loc
1	*CaPLATZ1*	*Capana01g001082*	1	29,898,510	29,900,326	−	231	25,991.48	9.30	nucl
2	*CaPLATZ2*	*Capana01g003577*	1	229,778,174	229,780,086	−	241	27,134.36	8.93	nucl
3	*CaPLATZ3*	*Capana01g004338*	1	296,584,241	296,586,323	−	182	20,484.66	9.73	nucl
4	*CaPLATZ4*	*Capana02g001147*	2	111,660,814	111,663,906	−	240	27,282.98	6.16	nucl
5	*CaPLATZ5*	*Capana04g000021*	4	366,427	367,644	−	242	27,830.56	8.25	nucl
6	*CaPLATZ6*	*Capana05g000900*	5	34,861,705	34,865,244	+	174	20,057.15	8.84	nucl
7	*CaPLATZ7*	*Capana06g001532*	6	36,555,377	36,556,697	−	245	27,769.75	9.30	nucl
8	*CaPLATZ8*	*Capana07g000312*	7	15,338,566	15,340,312	−	204	23,382.8	7.05	chlo
9	*CaPLATZ9*	*Capana07g001262*	7	168,116,678	168,126,780	+	189	21,752.29	8.75	cyto
10	*CaPLATZ10*	*Capana09g000109*	9	4,956,382	4,957,541	−	247	28,113.13	8.30	nucl
11	*CaPLATZ11*	*Capana10g001532*	10	163,187,426	163,189,479	+	204	22,819.09	8.97	nucl

Note: Loc, subcellular location.

**Table 2:** Characteristics of conserved motifs identified in pepper *PLATZ* proteins.

Motif	Length (aa)	Best Possible Match
1	41	HKDHRLJQIRRYVYHDVVRLNEIZKYJDCSSVQTYIINSAK
2	35	KPPWLKPLLKEKFFVACKIHEDAKKNEKNMYCLDC
3	29	KGVTNTCEICERSLLDSFRFCSLGCKVVG
4	17	NYKTMKRRKGIPHRAPL
5	11	VVFLNERPQPR
6	41	FLRRCTTLQLGPDFFIPNDMGDDDMANETAHSTIVDSDEPW
7	14	NKIQSFSPSTPPPT
8	11	NLALCPHCLSS
9	21	VMSFPCTEFVRKKRSGLHVCG
10	17	TSKNFVKKPKQSPEKKR
1	41	HKDHRLJQIRRYVYHDVVRLNEIZKYJDCSSVQTYIINSAK
2	35	KPPWLKPLLKEKFFVACKIHEDAKKNEKNMYCLDC
3	29	KGVTNTCEICERSLLDSFRFCSLGCKVVG
4	17	NYKTMKRRKGIPHRAPL

## Data Availability

Data are contained within the article and Supplementary Materials.

## Supplementary Materials

The following supporting information can be downloaded at: https://www.mdpi.com/article/10.3390/genes16060632/s1. Table S1 The identified amino acid sequences of PLATZ in Arabidopsis, pepper. Table S2 The primer sequences used in this study.

## References

[1] Van Tol N., Van der Zaal B.J. (2014). Artificial transcription factor-mediated regulation of gene expression. Plant Sci..

[2] Noman A., Aqeel M., Khalid N., Islam W., Sanaullah T., Anwar M., Khan S., Ye W., Lou Y. (2019). Zinc finger protein transcription factors: Integrated line of action for plant antimicrobial activity. Microb. Pathog..

[3] Samoluk S.S., Seijo G. (2025). Genome-wide analysis of the PLATZ gene family provides insights into the genome evolution of cultivated peanut (*Arachis hypogaea*, Leguminosae). Genet. Resour. Crop. Evol..

[4] Azim J.B., Khan M.F.H., Hassan L., Robin A.H.K. (2020). Genome-Wide Characterization and Expression Profiling of Plant-Specific PLATZ Transcription Factor Family Genes in *Brassica rapa* L.. Plant Breed. Biotechnol..

[5] Xu M., Gao Z., Li D., Zhang C., Zhang Y., He Q., Qi Y., Zhang H., Jiang J., Xu X. (2024). Functional prediction of tomato PLATZ family members and functional verification of SlPLATZ17. J. Integr. Agric..

[6] He X., Liu M., Fang Z., Ma D., Zhou Y., Yin J. (2021). Genome-wide Analysis of a Plant AT-rich Sequence and Zinc-binding Protein (PLATZ) in *Triticum Aestivum*. Phyton.

[7] Sun Y., Liu Y., Liang J., Luo J., Yang F., Feng P., Wang H., Guo B., Ma F., Zhao T. (2022). Identification of PLATZ genes in Malus and expression characteristics of MdPLATZs in response to drought and ABA stresses. Front. Plant Sci..

[8] Han X., Rong H., Tian Y., Qu Y., Xu M., Xu L. (2022). Genome-Wide Identification of PLATZ Transcription Factors in *Ginkgo biloba* L. and their expression characteristics during seed development. Front. Plant Sci..

[9] Zhao J., Zheng L., Wei J., Wang Y., Chen J., Zhou Y., Chen M., Wang F., Ma Y., Xu Z. (2022). The soybean PLATZ transcription factor GmPLATZ17 suppresses drought tolerance by interfering with stress-associated gene regulation of GmDREB5. Crop J..

[10] Kim J.H., Kim J., Jun S.E., Park S., Timilsina R., Kwon D.S., Kim Y., Park S., Hwang J.Y., Nam H.G. (2018). ORESARA15, a PLATZ transcription factor, mediates leaf growth and senescence in *Arabidopsis*. New Phytol..

[11] Yamada M., Han X., Benfey P.N. (2020). RGF1 controls root meristem size through ROS signalling. Nature.

[12] Iocco-corena P., Chaïb J., Torregrosa L., Mackenzie D., Thomas M.R., Smith H.M. (2021). VviPLATZ1 is a major factor that controls female flower morphology determination in grapevine. Nat. Commun..

[13] Wang A., Hou Q., Si L., Huang X., Luo J., Lu D., Zhu J., Shangguan Y., Miao J., Xie Y. (2019). The PLATZ Transcription Factor GL6 Affects Grain Length and Number in Rice. Plant Physiol..

[14] Hu Y., Liu Y., Lu L., Tao J., Cheng T., Jin M., Wang Z., Wei J., Jiang Z., Sun W. (2023). Global analysis of seed transcriptomes reveals a novel PLATZ regulator for seed size and weight control in soybean. New Phytol..

[15] González-morales S.I., Chávez-montes R.A., Hayano-kanashiro C., Alejo-jacuinde G., Rico-cambron T.Y., De folter S., Herrera-estrella L. (2016). Regulatory network analysis reveals novel regulators of seed desiccation tolerance in Arabidopsis thaliana. Proc. Natl. Acad. Sci. USA.

[16] Liu M., Wang C., Ji Z., Lu J., Zhang L., Li C., Huang J., Yang G., Yan K., Zhang S. (2023). Regulation of drought tolerance in *Arabidopsis* involves the PLATZ4-mediated transcriptional repression of plasma membrane aquaporin *PIP2;8*. Plant J. Cell Mol. Biol..

[17] Zhang K., Lan Y., Wu M., Wang L., Liu H., Xiang Y. (2022). PhePLATZ1, a PLATZ transcription factor in moso bamboo (*Phyllostachys edulis*), improves drought resistance of transgenic *Arabidopsis thaliana*. Plant Physiol. Biochem..

[18] Zhang S., Yang R., Huo Y., Liu S., Yang G., Huang J., Zheng C., Wu C. (2018). Expression of cotton PLATZ1 in transgenic *Arabidopsis* reduces sensitivity to osmotic and salt stress for germination and seedling establishment associated with modification of the abscisic acid, gibberellin, and ethylene signalling pathways. BMC Plant Biol..

[19] Zhang Z., Zhang J., Wang C., Chang Y., Han K., Gao Y., Xie J. (2024). Characterization of GPX Gene Family in Pepper (*Capsicum annuum* L.) under Abiotic Stress and ABA Treatment. Int. J. Mol. Sci..

[20] Qin C., Yu C., Shen Y., Fang X., Chen L., Min J., Cheng J., Zhao S., Xu M., Luo Y. (2014). Whole-genome sequencing of cultivated and wild peppers provides insights into Capsicum domestication and specialization. Proc. Natl. Acad. Sci. USA.

[21] Kumar S., Stecher G., Li M., Knyaz C., Tamura K. (2018). MEGA X: Molecular Evolutionary Genetics Analysis across Computing Platforms. Mol. Biol. Evol..

[22] Chen C., Wu Y., Li J., Wang X., Zeng Z., Xu J., Liu Y., Feng J., Chen H., He Y. (2023). TBtools-II: A one for all, all for one bioinformatics platform for biological. Mol. Plant.

[23] Lescot M., Déhais P., Thijs G., Marchal K., Moreau Y., Van de peer Y., Rouzé P., Rombauts S. (2002). PlantCARE, a database of plant cis-acting regulatory elements and a portal to tools for in silico analysis of promoter sequences. Nucleic Acids Res..

[24] Liu F., Yu H., Deng Y., Zheng J., Liu M., Ou L., Yang B., Dai X., Ma Y., Feng S. (2017). PepperHub, an Informatics Hub for the Chili Pepper Research Community. Mol. Plant.

[25] Xianyang L., Fei H., Guoqing Z., Mingna L., Ruicai L., Junmei K., Qingchuan Y., Lin C. (2023). Genome-Wide Identification and Phylogenetic and Expression Analyses of the PLATZ Gene Family in *Medicago sativa* L.. Int. J. Mol. Sci..

[26] Jiahui Q., Hui W., Xinyi W., Muhammad N., Ya W., Dayong L., Fengming S. (2023). Genome-wide characterization of the PLATZ gene family in watermelon (*Citrullus lanatus* L.) with putative functions in biotic and abiotic stress response. Plant Physiol. Biochem. PPB.

[27] Feng X., Zhu G., Meng Q., Zeng J., He X., Liu W. (2024). Comprehensive analysis of PLATZ family genes and their responses to abiotic stresses in Barley. BMC Plant Biol..

[28] Li J., He W., Dai Z., Xie D., Sun J. (2024). Genome-Wide Analysis of the PLATZ Gene Family and Identification of Seed Development-Related Genes in Flax [*Linum usitatissimum* L.]. J. Nat. Fibers.

[29] Shen C., Du H., Chen Z., Lu H., Zhu F., Chen H., Meng X., Liu Q., Liu P., Zheng L. (2020). The Chromosome-Level Genome Sequence of the Autotetraploid Alfalfa and Resequencing of Core Germplasms Provide Genomic Resources for Alfalfa Research. Mol. Plant.

[30] Wang Z., Hobson N., Galindo L., Zhu S., Shi D., Mcdill J., Yang L., Hawkins S., Neutelings G., Datla R. (2012). The genome of flax (*Linum usitatissimum*) assembled de novo from short shotgun sequence reads. Plant J. Cell Mol. Biol..

[31] Cannon S.B., Mitra A., Baumgarten A., Young N.D., May G. (2004). The roles of segmental and tandem gene duplication in the evolution of large gene families in *Arabidopsis thaliana*. BMC Plant Biol..

[32] Zhang L., Yang T., Wang Z., Zhang F., Li N., Jiang W. (2023). Genome-Wide Identification and Expression Analysis of the PLATZ Transcription Factor in Tomato. Plants.

[33] Zhao D., Chen P., Chen Z., Zhang L., Wang Y., Xu L. (2023). Genome-wide analysis of the LBD family in rice: Gene functions, structure and evolution. Comput. Biol. Med..

[34] Yamaguchi-shinozaki K., Shinozaki K. (2005). Organization of cis-acting regulatory elements in osmotic- and cold-stress-responsive promoters. Trends. Plant Sci..

[35] Huang J., Zhao X., Bürger M., Chory J., Wang X. (2023). The role of ethylene in plant temperature stress response. Trends. Plant Sci..

[36] Raghunath A., Sundarraj K., Nagarajan R., Arfuso F., Bian J., Kumar A.P., Sethi G., Perumal E. (2018). Antioxidant response elements: Discovery, classes, regulation and potential applications. Redox Biol..

[37] Ahluwalia O., Singh P.C., Bhatia R. (2021). A review on drought stress in plants: Implications, mitigation and the role of plant growth promoting rhizobacteria. Resour. Environ. Sustain..

