# Supplementary material for: Genome-Wide Identification of CaPLATZ Family Members in Pepper and Their Expression Profiles in Response to Drought Stress
Source: Genes (Basel). 2025 May 24;16(6):632. doi: 10.3390/genes16060632 (PMC12191615; doi:10.3390/genes16060632)
Supplement: Supplementary file 1 [file genes-16-00632-s001.zip › genes-3655414-supplementary.pdf]

Table S1 The identified amino acid sequences of PLATZ in Arabidopsis, pepper.

>AtPLATZ1

MGPMIRTEEEEDYTSPPWLMPMLRGSYFVPCSIHVDSNKNECNLFCLDCAGNAFCSYCLVKHKDHRVVQIRRS
SYHNVVRVNEIQKFIDIACVQTYIINSAKIVFLNERPQPRIGKGVTNTCEICCRSLLDSFRFCSLGCKLGGMRRGDL
SLTFSLKGKHGREYLGGSESDEATTPTKMRKTNAFNRLMSGLSISTVRFDDYGPNGDQRSSSSGDEGGFSFSPG
TPPIYNHRNSSRRKGVPHRAPF

>AtPLATZ2

MVREGEEEEEMMMMMATKPAWLEGLMAETFFSSCGIHETRRKSEKNVFCLLCCLSVCPHCLPSHRSHPLLQV
RRYVYHDVVRLSDLEKLIDCSYVQPYTINGAKVIFLNQRQQSRAKVSSNVCFTCDRILQEPFHFCSLSCKVDYLSY
QGDDLSSILYRIDESDFTFEGLRMDGHDQLGEISTMEDGEDILVISDESEQGNNSHKKEKKKSKKKKPESNYLPG
MVLSSLGNRRKGAPHRAPFS

>AtPLATZ3

MGAEEETNKTYPHWLKPLLREKFFVQCKLHADSHKSECNMYCLDCTNGPLCSLCLSFHKDHHAIQIRRSSYHD
VIRVSEIQKFLDITGVQTYVINSAKVVFLNERPQPRPGKGVINTCEVCYRSLVDSFRFCSLGCKISGISKKKRKEWT
NNLSDSDDSYSSTSIGRLKKNDDIMNNSFTPSTPPLSAVNRRIAKRRKGIPHRAPFGGLIIEY

>AtPLATZ4

MENDDVMTPPWLTPMLRADYFVTCSIHSQSSKSECNLFCLDCSGNAFCSSCLAHHRTHRVIQIRRSSYHNVVR
VSEIQKHIDISCIQTYVINSAKIFFLNARPQCRTGKSLNKTCQICSRNLLDSFLFCSLACKLEGVKNGEDPNLTLFHS
GKSDDSSKIINTGICSRLIDGISIAVDDQRSETAGVLSPETPSIESHRNYPMKSRRKGIPQRAPF

>AtPLATZ5

MGPMMMRAEEDNYNLNPPPWLIPMLRANYFIPCSIHAASNKSECNMFCLDCSSEAFCSYCLLNHRNHRVLQI
RRSSYHNVVRVNEIQKYIDISCVQTYIINSARIVFLNERPQPRIGKGVTNTCEICCRSLLDSFRFCSLGCKLGGMKR
DPSLTFSLRGKHGREYEGEWESDEATTPTKIRKTCAFNRLMSGLSISTVKCDYLSGDQPSSSSGDESGFKLSPGTP
PIYNHRNSSRRKGVPHRAPF

>AtPLATZ6

MNLSEKRRSEEVWIETLLNSEFFGICMNHKYLRKNEKNVFCIDCNVEICRHCCNTVTDSHFLHRRLQICKYVYQ
DVIRLLEIQNYFDCSEIQTYKINGEKAIHLNSRPQAKDARPSTKAKNGASCVTCKRYIQDHPNLFCSISCKISTPSK
KHKFCFSPKLEQSVLEKEHSTQEGSLEEKKSCTSSLTDVSEDSEVLLSDFSFRPLLRILKRKGISRRSPFY

>AtPLATZ7

MGIQKPAWLDALYAEKFFVGCPYHETAKKNERNVCCLDCCTSLCPHCVPSHRFHRLLQVRRYVYHDVVRLEDL
QKLIDCSNVQAYTINSAKVVFIKKRPQNRQFKGAGNYCTSCDRSLQEPYIHCSLGCKVDFVMKRYRDITPFLKPC
HTLTLGPDYIIPQDLLTDDEVAAYETPRSTVVDGDESMSWSSASSDNNNAGAAAAYAATTTHVVRKKRTGFCLC
AKSANSYKEVSEDPDDISACINRRKGVPQRSPLC

>AtPLATZ8

MEEPKWLEGLLRTNFFSICPRHRETPRNECNMFCLSCQNAAFCFYCRSSFHIDHPVLQIRRSSYHDVVRVSEIEN
ALDIRGVQTYVINSARVLFLNERPQPKNSSHGAASSTPKTMSYFCETCCRTLLDPFRFCSLGCKVEGMRKNKEEE
EERLRKERQQETHKGTHPPTHTSNSRRRKGIPHRAPFAS

>AtPLATZ9

MSQYMDISGIHLYSINGFPIVYINQRRGNNNHRSRSNVMHKCKICEWEIDAASSALFCSMECKFRSVLGSQLDE
LMENSSEVTEISEEIDEPVMKKRHRRKGSPHRAPFF

>AtPLATZ10

MESGEFPAWLEVLLKDKFFNACLDHEDDKKNEKNILCIDCCLTICPHCLSSHTSHRLLQIRRYVYRDVLRVEDGSK
LMDCSLIQPYTTNSSKVVFINERPQSRQFRGSGNICITCDRSLQSPYLFCCLSCKISDVIMRQRGLSGFLRVCNVL
DLTDEVTTTTPSSTLEPTGSNRTSSESSGNEGEDMFWCQALACTATTEIVRKKRSSLSTTCRRVTEVVSTTNTEAP

VNFLNRRKNPPQRAPLY
>AtPLATZ11
MAIEDQENTIREIKPKNRRIMGAGGPEEEENRWPPWLKPLLKEQFFVHCKFHGDSHKSECNMYCLDCTNGPL
CSLCLAHHKDHRTIQIRRSSYHDVIRVNEIQKYLDIGGIQTYVINSAKVVFLNERPQPRPGKGVTNTCKVCYRSLV
DDSFRFCSLGCKIAGTSRGFEKGRENLLMETEDSSSSIAIGKNITNLQSFSPSTPPLTTSSNCRIVKRRKGIPHRSP
MG
>AtPLATZ12
MAIEDYENPNREIKPKNRRFMEGENQWPIWLKPLLNQHFFAQCKFHGHLPRTECKMYCLDCTNDSFCSLCLSE
HENHRTIQIRISSYHNVTKVDEIQKYLDISSIQTYVINSSKVLFLNERPQSKPGKGFTNACMVCYRGLAENCFRFC
SIGCKVAGTSGVFQKRVKHTTNDSDNSNNSSGVENNSSGAENGNSNLQSLSPPTPQFPPRSLRKRLRKGIPHR
APFS
>CaPLATZ1
MGAGGPDEEENKWPPWLKPLLKERFFVQCKLHADSHKSECNMYCLDCMDGALCSLCLSHHKDHRAIQIRRSS
YHDVIRVNEIQKYLDITSVQTYIINSAKVVFLNERPQPRPGKGVTNTCEVCERSLLDSFRFCSLGCKIVGTSKNFVK
RPKQSPEKKRQSPVAASSSYDSEDSYSSSSHHHGQQNSPSSNKVQSFSPSTPPPTSVNYRTAKRRKGIPHRAPM
GGHLVIGY
>CaPLATZ2
MMLDYTQHLPQWLGALLSEKFFNACIIHEDAKKNEKNVFCLDCCEGLCPRCLNTHRSHRLLQIRRYVYHDVIRL
GDANKLLDCGFVQSYITNSAKVIFLNQRPQMRASRYSGNCCIICDRGLQDPFLFCSVSCKVQHILKTEGELTKYIY
RCTYMTLPEPGLDDGQMTPDTILEPVGSTRSESGSSGGEVGCRAVLCTAATEIVRKKRSTLSAFRSVFRPACGPG
SGMNRRKGTPQRSPLY
>CaPLATZ3
MYCLDCINGPLCSLCLSHHKDHIAIQIRRSSYHDVIRVNEIQKYLDISSVQTYIINSAKVVFLNERPQPRPGKGVTN
TCQVCERSLLDSFKFCSLGCKVIGTSKNFVKKLRQLSTKRRLTMATSDSDDSCSSSSHRGYKSAINKIQSFIPSTPPP
TSANYRTTKRRKGIPHRSPMGGLLIEY
>CaPLATZ4
MEEMKPAWLEGLMAEKFFAGCGVHQNRRKNEKNIFCLECCQSFCPHCLPQHHSHPLLQVRRYVYQDVIRLDD
LEKLIDCSYIQPYTINSAKVIFLNQRAQSRSCKVSGNSCFTCDRVLQDPFNFCSLSCNVDYMVYQGEDLSNILYKF
DDSEFALSQFEGLHVDTSDLIDEESQITPNSILEDPLECRGSSCSNNIMDNSGISHGGRMIKNKKKGSGFFPGLVL
SLNNRRKGAPQRSPLS
>CaPLATZ5
MVSQVRKDEIGPAWLKPLLKASYFHTCALHADSNKSECNMYCLDCMGTALCSYCLIKHKDHRLVQIRRSSYHNV
VRVNEIQRYIDISCIQTYIINSAKIVFLNERPQPRPGKGVTNTCEICARSLLDSFRFCSLGCKLNGIRRGDEELTFAVK
AKTNQGFEYSDESSTTPKKKQRRDDRVMFGFRHLNIEEGTTNSDGEEEEEEEEEAANLHMIYSMSPDTPPIYN
HRNSSRRKGIPHRAPF
>CaPLATZ6
MFCIDCNLCFCKHCVSSSSHCFHEWLQICKYVYHDVVRLHEIQKYLNCSEIQTYKINGEKAVHLNPRPQSKDSKT
SKLKGSVTCEACGRHLQDLPNRFCSIACKVSIDANICKEHYQKNFVSTQITKFDLSNENESCISLNESSEVIQTWCI
STLKPRKNLHKRKGVPRRAPIC
>CaPLATZ7
MLFHSFICSNFSKNLNSSASWLPELSPLLGPDEDDNRWPPWLKPLLNERFFVQCKLHADSHKSECNMYCLDCI
NAPLCSLCLAHHKDHRVIQIRRSSYHDVIRVNEIQKYLDISSVQTYIINSAKVVFLNERPQPRPGKGVTNTCDVCE
RSLLDSFKFCSLGCKIVGTSNNFVKKAKNSQEKKRSPVKALESEDSYSSSSSQGRLNKIKSFTPSTPPPTSANYKTS
KRRKGIPHRAPTGGLFIEY

>CaPLATZ8
MGIQKPAWLEALYREKFFAACSIHEGAKKNEKNICCLDCCISICPHCVMAHRFHRLLQIRRYVYHDVVRLEDLEK
LIDCSNVQVDFVIKHYKDISPFLRRCTTLQLGPDFFIPNDMTDDDTANETAHSTIVDSDEPWGSSTSGSSGSEN
MMMNFPCTEFVRKKRSGLYVCGRITLNSYKNITDNEDMATSMSRRKGIPHRSPLC
>CaPLATZ9
MASSKSSAFETIEQEELVEEPMRNMPPWLGPFLKKTYFGSCLVHDELQKNDLNKYCITCDLDLCRYCISTDKHND
HELLKIHRHVYKDVVPLEQMENHIDCAKIQPYKCNKKWVIALNPLPHCGSGSLIAGDPTCYTCKRRLNDPGQFS
FCSIACQVEAVCRKYGISLGAIEMKRKRKRKGIPRRAPFQ
>CaPLATZ10
MGIHKPLWLKALYVEKFFVPCSIHESAKKNEKNVCCLDCNISICPHCVTSHRVHRLLQIRRYVYHEVVRLEDLENL
IDCSNIQAYTINNAKVVFIKKRPQNRQFKGGNYCTSCDRSLQEPFVHCSLGCKVDFVLKHHKDLSPFLRRCTTLQ
LSSDFFIPQDMGDDDMAIETAHSTVVDSDEPWSSSSSTGSGSENMSFPCTEFVRKKRSGMHVCGRSANNCN
NVAEEEDMATSMSRRKGIPQRSPLC
>CaPLATZ11
MTMLVPPWLEPLLNTDFFSICRTHGDAARSECNMYCLDCNDNAFCFYCRSSKHKDHQVIQIRRSSYHDVVRVS
EIQKVLDISGVQTYVINSARVLFLNERPQPKSSGKASSHVCEICGRSLLDTFRFCSLGCKLVGIKRNGDASFILDAK
NEVIALQRGEGISSRGGNQLREGLEHDIYPTTPPPPPSNARRRKGIPHRAPLGS

Table S2 The primer sequences used in this study

Gene ID	Primer sequence F (5'-3')	Primer sequence R (5'-3')
β-Actin	CCACCTCTTCACTCTCTGCTCT	ACTAGGAAAAACAGCCCTTGGT
Capana01g001082	GCGGCCTCCTCCTCCTAT	CAGAAGTTGGAGGGGGCG
Capana01g003577	TGGAGGAGAGGTGGGGTG	GTCCACAAGCCGGTCGAA
Capana01g004338	CAGTAGTAGCCACCGCGG	CAGAAGTTGGAGGGGGCG
Capana02g001147	TGCAGGATGTGGGGTCCA	GTGGAAGGCAGTGAGGGC
Capana04g000021	GCGAGGCGATGAAGAGCT	TCTGTCGTCCCTGCGTTG
Capana05g000900	TGAATCCACGTCCTCAATCGA	ATGTCTTCCACAGGCTTCACA
Capana06g001532	TTCACTCCGTCAACGCCC	GTCCTCCTGTTGGGGCAC
Capana07g000312	TGACCGATGATGACACAGCT	CGGATGTCGATGAGCCCC
Capana07g001262	TGCATGCCAGGTGGAAGC	CTTCCTCTTGCGCTTGCG
Capana09g000109	TGGGCGACGATGACATGG	TCCTGAGCCCGTGGATGA
Capana10g001532	ATGCTGGTTCCACCGTGG	ATTCACTCCGCGCTGCAT
